# Reproductive agency and the acceptability of divorce, abortion, and homosexuality among migrants from the Middle East and Africa living in Sweden–a cross-sectional analysis

**DOI:** 10.1186/s12939-025-02400-x

**Published:** 2025-02-24

**Authors:** Mia L. van der Kop, Karin Båge, Veronika Tirado, Anna Kågesten, Bi Puranen, Rachael Sorcher, Anna Mia Ekström, Elin C. Larsson

**Affiliations:** 1 Department of Global Public Health, Karolinska Institutet, Widerströmska Huset, Tomtebodavägen 18A, Stockholm, SE-171 77 Sweden; 2 World Values Survey Association, Stockholm, Sweden; 3 Institute for Futures Studies, Stockholm, Sweden; 4 Department of Infectious Diseases/Venhälsan, South General Hospital, Stockholm, Sweden; 5 Department of Women’s and Children’s Health, Karolinska Institutet, Stockholm, Sweden

**Keywords:** Health, Migrants, Sweden, Sexual and reproductive health and rights, Reproductive agency, Social norms

## Abstract

**Background:**

Sweden has a longstanding history of promoting sexual reproductive health and rights. Reproductive decision-making is a fundamental right, but an individual’s decision-making power differs across contexts. We examined self-reported reproductive agency and the acceptability of divorce, abortion and homosexuality among migrants in Sweden originating from the Middle East or North Africa (MENA) and Sub-Saharan Africa (SSA).

**Methods:**

This cross-sectional study used face-to-face interview data from the 2018–2019 Migrant World Values Survey (MWVS) and included individuals 18–49 years old who migrated to Sweden from MENA or SSA. Partial proportional odds models were used to estimate adjusted odds ratios (aOR) and corresponding confidence intervals (CI) of associations between sociodemographic factors and two outcomes: 1) reproductive agency (decision-making power on when, with whom, and how many children to have), measured on a 10-point scale categorized as low (1–4), moderate (5–7), and high (8–10); and 2) the Choice Sub-Index (CSI), a composite index of the acceptability of divorce, abortion, and homosexuality, categorized as 0- < 0.4 (low), 0.4- < 0.7 (moderate), and 0.7–1.0 (high).

**Results:**

Between September 2018 and November 2019, 7991 participants responded to the MWVS, of whom 4669 met the inclusion criteria. Almost 3/4 (73%) of respondents expressed a high degree of reproductive agency, but less than five per cent of respondents had a high value on the CSI. Living in Sweden ≥ 4 years was associated with higher values on the CSI (aOR 1.76, 95% CI 1.15–2.67), while identifying as Muslim was associated with having a low value on the CSI (aOR 0.44, 95% CI 0.32–0.63). Neither duration of time in Sweden nor identifying as Muslim were associated with reproductive agency. Age and reason for migration (family reunification or as a refugee) were not associated with either outcome.

**Conclusion:**

Our study found that migrants from MENA and SSA expressed a high degree of reproductive agency. Migrants had low values of a combined measure of the acceptability of divorce, abortion and homosexuality; however, acceptance increased with time spent in Sweden. Understanding factors associated with migrants’ sense of reproductive agency and their values and how these change over time in Sweden provides a foundation for working towards equitable sexual and reproductive health and rights.

**Supplementary Information:**

The online version contains supplementary material available at 10.1186/s12939-025-02400-x.

## Introduction

Sexual and reproductive health and rights (SRHR) are fundamental human rights, yet no country has succeeded in fully realizing SRHR for all. Furthermore, they are not equitably fulfilled globally and under pressure in many contexts [1–6]. SRHR is also an integral part of the United Nations 2030 Agenda and its Sustainable Development Goals (SDG). SDG 3, Good Health and Well-being, includes targets to integrate reproductive health into national strategies and programmes. SDG 5, Gender Equality, aims to provide universal access to sexual and reproductive health [7, 8]. Despite progress in improving SRHR globally, barriers still exist and include a lack of political will, legal reform, economic capital, and social norms surrounding SRHR [9, 10]. These barriers often impact those already marginalized, such as migrants, who are least likely to benefit from SRHR policies and programs [7, 10]. Migrants, including asylum seekers and refugees, often experience SRHR inequalities both at the healthcare system and the patient-provider levels. Disparities also exist in migrants’ health-seeking behavior and the perceived cultural and social freedom to make one’s own choices [11–13]. Disparities may be due to social and financial vulnerabilities, restricted access to healthcare because of language barriers, discrimination, and a lack of knowledge of how to navigate the healthcare system [14, 15]. These factors may negatively impact migrants’ decision-making over their reproductive and sexual lives [13, 16].

A systematic review of 28 studies published between 2000 and 2020 in several different countries found that social norms towards sexual and reproductive health, often grounded in socio-cultural and religious traditions, may impede migrants’ access to and fulfillment of SRHR in the country of destination [11]. While social norms and values regarding SRHR vary across populations, cultures, and countries, they consistently play a crucial role in shaping individuals’ perspectives, knowledge, choices, and behaviors related to relationships, intimacy, and family [9, 12]. For example, a study of migrants in Sweden conducted between 2018 and 2019 found that social norms that stigmatize abortion were associated with limited knowledge of abortion law [17]. 2016 data from Norway indicates youths with origins outside of Western Europe are less accepting of homosexuality; however, they consider themselves to have more positive attitudes than their parents [18]. In Syria, divorce laws are highly discriminatory towards women. For many Syrian refugee women living in Germany, the ‘gains’ of a divorce in Germany exceed the ‘gains’ of remaining married. At the same time, Syrian refugee men tend to interpret the Islamic meaning of marriage the same way that they did in Syria [19], which illustrates how socio-cultural and religious norms may be brought forward to a destination country. Social norms among migrants’ origin countries may impact their acceptance of divorce, abortion, and homosexuality, as well as their sense of reproductive agency, or the capacity to make free choices over one’s body including reproductive decisions—a crucial aspect of women’s empowerment and a prerequisite for fulfilling SRHR [10, 20, 21].

Findings from longitudinal global studies of social norms and values related to SRHR have found that Sweden is a clear outlier in terms of its supportive norms [9, 21]. The World Values Survey (WVS) indicates that Sweden had the greatest score of all countries that score highly in secular-rational and self-expression values [22]. A high score reflects a prioritization of individualism over social conformity, a global rather than nationalistic outlook, an emphasis on subjective well-being, a high level of interpersonal trust, and acceptance of homosexuality and abortion [22]. These norms are reflected in Sweden’s laws, which legalized abortion on request and criminalized marital rape in the 1970s, enacted marriage equality in 2009, and in 2018, updated its law on sexual consent to include non-verbal cues [17, 23].

Sweden also stands out as having the highest number of refugees per capita in the Europe [24]. Most originate from the Middle East and North Africa (MENA) e.g. Iran, Iraq, Syria, Afghanistan, Somalia, Eritrea, Turkey; as well as Sub-Saharan Africa (SSA) e.g. Sudan and the Democratic Republic of the Congo [25]. While these countries’ policies on SRHR vary, their norms and legislation related to SRHR are generally more restrictive than in Sweden. However, reproductive agency and individuals’ values are amenable to change over time, and following migration to another environment, behaviors and norms may shift to become more similar to that of the majority population [26].

Migrants moving to Sweden from countries where norms greatly differ might experience challenges in understanding and navigating their new context, which may affect their SRHR. Little is known at a granular level about MENA and SSA migrants’ sense of reproductive agency and emancipative values after moving to Sweden. Results from the Migrants WVS (MWVS) [27] showed that more years lived in Sweden was associated with increased values on the Choice Sub-Index (CSI), an index measuring the acceptability of divorce, abortion and homosexuality, although confounding variables were not considered. The same report found that migrants from Turkey, Iran, and Iraq in Sweden scored higher on the CSI compared to those still living in their countries of origin, but not as high as the Swedish majority population [27]. Another study using WVS data found that only 53% of women and men in Ethiopia, Kenya, and Zimbabwe highly rated their sense of choice and control over whether, when, and how many children they wish to have (median 9 on a 1–10 scale) [28]. Despite these important overviews, there is limited understanding of the factors associated with migrants’ sense of reproductive agency and acceptance of homosexuality, abortion and divorce. Such information is critical to understanding and effectively addressing the prevailing SRHR inequalities between migrants and non-migrants living in Sweden. Examining migrants’ sense of reproductive agency and how acceptable they find divorce, abortion and homosexuality can help determine the gap between those born in Sweden and those born outside of Sweden, with the goal of achieving equity and ensuring SRHR for all.

### Objectives

This study uses MWVS data to assess self-reported reproductive agency and the acceptance of divorce, abortion and homosexuality among migrants from MENA and SSA living in Sweden. Our objectives were to: 1) determine socio-demographic factors associated with reproductive agency; and 2) determine socio-demographic factors associated with values on the CSI (a composite index measuring the acceptance of divorce, abortion and homosexuality).

### Theoretical grounding

While its definitions vary, reproductive agency can be thought of as an integral component of reproductive empowerment and conceptualized as “being able to set individual reproductive goals and follow through with actions to realize the goals” [21]. We focused on the individuals’ *sense* of reproductive agency, defined as being able to decide whether, when, and with whom to have children [21].

To measure the acceptance of divorce, homosexuality and abortion, we used the CSI developed by Welzel. ‘Choice’ is one of four parts of an index on emancipative values and is defined as a measure of how strongly people value agency in their reproductive choices. ‘Choice’ is considered an emancipative value with a liberating orientation [22]. Both agency and values are influenced by socio-cultural norms, which in the case of migrants, include cultural norms of their country of origin, the migrant community if applicable, and those of the host country [9, 29–31].

## Methods

### Study design, setting, and participants

The WVS is a global survey that collects data on people’s values and norms every five years since 1981. Using face-to-face interviews, it has collected data in over 140 countries representing more than 90% of the world’s population. All of the WVS data is accessible on its website [22]. This cross-sectional study used the Swedish Migrant WVS (MWVS). The MWVS in Sweden is based on the standard WVS but also includes items on how values and social norms change when moving from one country to another as well as additional questions focusing on perceptions related to various dimensions of SRHR.

The MWVS sample included 7,991 migrants who were interviewed in parallel with the seventh WVS wave in Sweden. Statistics Sweden's data were used to obtain a list of the population, and principles decided on by the Municipal Assemblies in European Local Governance (MAELG) were used to select municipalities from which to draw participants [32]. The sampling procedure is described in the Online Supplementary Material, additional file 1. For Sweden, 54 of 290 municipalities were selected, representing 20% of Sweden’s population. The municipalities were intentionally chosen to be representative of the migrant population in Sweden (see additional file 1).

The MWVS questionnaires were administered face-to-face across Sweden between September 19, 2018, and November 27, 2019. Participants completed the questionnaire in the municipality in which they were registered and in the language with which they were most comfortable (Arabic, Dari, English, Somali, Swedish, Tigrinya, and Turkish). Most interviews were conducted at Swedish for Immigrants (SFI) schools, within the general school system, at workplaces, or within various civil organizations. The background variables of the respondents (sex, age, and country of origin) were then compared with national population characteristics of non-European migrants from each country of origin that was included, i.e., the seven largest countries of origin of migrants in Sweden. If any group of respondents deviated, weighting was used to ensure they were representative of their specific group. We randomly selected one class in each of four levels at SFI schools in which we interviewed everyone. Our response rate at the SFI schools was 98%. To recruit participants who had lived longer in Sweden, we sent postal invitations to take part in the survey, after which we interviewed them at a local library or a place of their choosing (for detail see Online Supplementary Material, additional file 1).

**Fig. 1:**
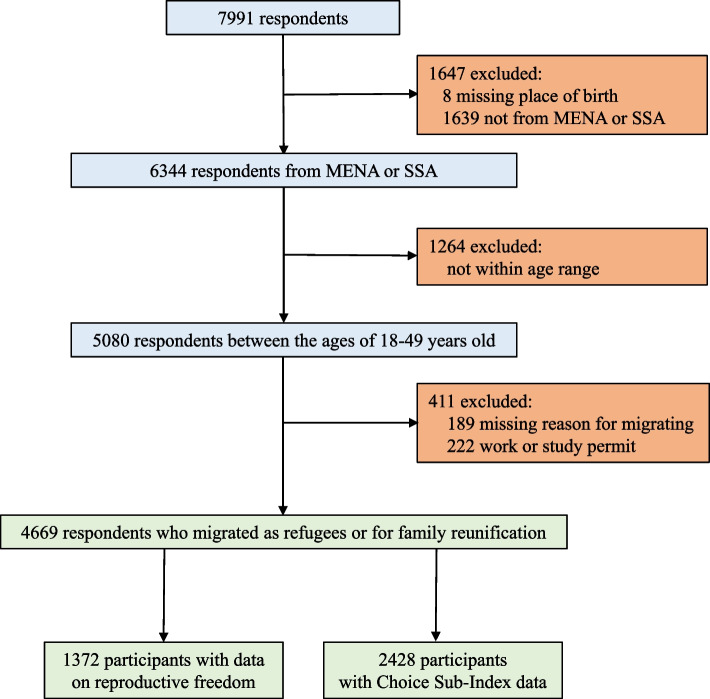
Participant flow diagram

The study population (*N* = 4669) included in this analysis are men and women between the ages of 18 and 49 (reproductive age) who were born outside of Sweden and who had migrated to Sweden from MENA or SSA as refugee or for family reunification (the first person had to have been granted a residence permit as a refugee) (Fig. 1).

### Variables

#### Outcome variables

In this study we measured individuals’ subjective assessments of their perceived reproductive agency through a single-item question: “How much can you decide yourself when it comes to your reproductive health (i.e., how many children you want, when and with whom)?” Responses were on a 10-point scale (1 = not at all, 10 = a great deal). Respondents could also indicate ‘Don’t know or want to answer’. Responses 1 to 4 were considered as having low reproductive agency, 5 to 7 moderate reproductive agency, and 8 to 10 a high level of reproductive agency.

The acceptance of divorce, abortion and homosexuality was measured using the WVS CSI, a sub-index of the broader emancipative values index (EVI), which has been validated in both Western and non-Western settings [22]. The CSI measures “how strongly people value freedom in their reproductive choices” by examining how acceptable participants find divorce, abortion, and homosexuality [33]. Questions were asked as follows: “Please tell me for each of the following actions whether you think it can always be justified, never be justified, or something in between.”. Responses were on a 10-point scale (1 = never justifiable, 10 = always justifiable). When participants answered all three components, the CSI value was an average of the three. If one of the components was missing, a linear transformation of the two available components was used. If only one value of the components was available, the CSI score was considered missing. CSI values range from a minimum value of 0 to a maximum value of 1.0, which we categorized into low (0- < 0.4), moderate (0.4- < 0.7) and high (0.7–1.0).

#### Covariates

We included the following sociodemographic covariates: age in years (continuous) and sex (male; female). Participants could also choose to define themselves as ‘other’ gender; however, this category was not included in the analysis due to small numbers (*n* = 12). Region of birth was based on World Bank criteria and categorized as i) MENA and ii) SSA. Most of the participants from MENA originated from Syria, Afghanistan, and Iraq; while most from SSA were born in Eritrea, Somalia and Ethiopia. This is reflective of the countries of birth of migrants living in Sweden from both MENA and SSA. An additional file shows the number of participants from each country (see additional file 2). Other variables include the participants’ identification as Muslim (yes; no); level of education (≤ secondary; post-secondary); employment (employed; unemployed; student); and marital status (married or cohabiting; single; separated, divorced or widowed). We also considered the number of children a participant had (continuous); how long the participant had lived in Sweden in years (≤ 1, 2–3, ≥ 4); and the reason for moving to Sweden (family reunification; refugee).

In addition, we examined the association between each outcome and the respondents’ value of gender equality, which is also one of the emancipative values from the WVS. We did not examine equality as an outcome, but rather as a factor potentially associated with CSI values or reproductive agency. The gender equality sub-index is based on answers to three statements: i) “A university education is more important for a boy than for a girl”; ii) “When jobs are scarce, men should have more right to a job than women”; and iii) “On the whole, men make better political leaders than women do.” Possible answers to these questions were strongly agree, agree, disagree, or strongly disagree. The equality sub-index was created similarly to the CSI. Equality’s maximum value is 1.00, which is the strongest possible emphasis on gender equality, and the minimum value is 0.00, representing the lowest possible emphasis on equality. Equality was categorized into low (0- < 0.4), moderate (0.4- < 0.7) and high (0.7–1.0).

### Statistical methods

Purposefully selected variables were examined using standard contingency tables, including frequencies and percentages. Pearson chi-square tests were used to test the significance of associations between categorical variables and levels of reproductive agency and CSI values. Histograms and measures of skewness and kurtosis were used to assess the normality of continuous variables before summarizing the data using means (standard deviations [SD]) and testing associations with an analysis of variance test. For non-normally distributed variables, medians (interquartile range [IQR]) are presented and the Kruskal–Wallis test was used. The number of participants who answered the survey during the study period determined the sample size.

We used a multivariate ordinal logistic regression model to examine associations between demographic characteristics and reproductive agency and CSI values. Separate models were built for each outcome, and all participants who had a value for each outcome were included in the respective model. For both models, we used the lowest level of the outcome as the reference level. To test the parallel regression assumption in the multivariate model, we used the likelihood ratio and Brant tests. These tests indicated that the parallel lines assumption was violated; therefore, we used partial proportional odds models, in which the parallel assumptions were relaxed only for explanatory variables where it was not justified. Where it was not justified, the results are presented in two panels. The first panel contrasts category 1 (low values of reproductive agency/CSI) with categories 2 (moderate values of reproductive agency/CSI) and 3 (high values of reproductive agency/CSI). The second panel contrasts categories 1 and 2 with 3. Adjusted odds ratios (aORs) and 95% confidence intervals (CI), together with *p*-values are presented. Pairwise deletion was used to handle missing data. For each outcome, we compared missingness based on the following variables: age, sex, education, region, and reason for coming to Sweden. Analyses were conducted using StataCorp. 2021. *Stata Statistical Software: Release 17.* College Station, TX: StataCorp LLC.

### Ethics

Ethical approval was received from the Swedish Ethical Review Authority. Informed consent was obtained from all respondents ahead of their participation.

## Results

All 7991 participants who completed the MWVS-7 in Sweden were screened for study eligibility. Of these participants, 4669 aged between 18 and 49 years from the MENA and SSA region who came to Sweden as a refugee or for family reunification were included (Fig. 1). An additional file presents descriptive characteristics (see additional file 3). Overall, the mean age of participants was 31 years (SD 9.10). Most participants were female (54.0%) and married or living with a partner (63.0%). Of the 4003 (85.7%) respondents who answered whether they had children, 59.9% of them did. Three-quarters of the respondents were born in the MENA (75.4%). Two-thirds of participants moved to Sweden as refugees (67.2%), and the median length of time respondents had lived in Sweden was three years (IQR 2–4).

### Association between socio-demographic characteristics and reproductive agency

**Fig. 2:**
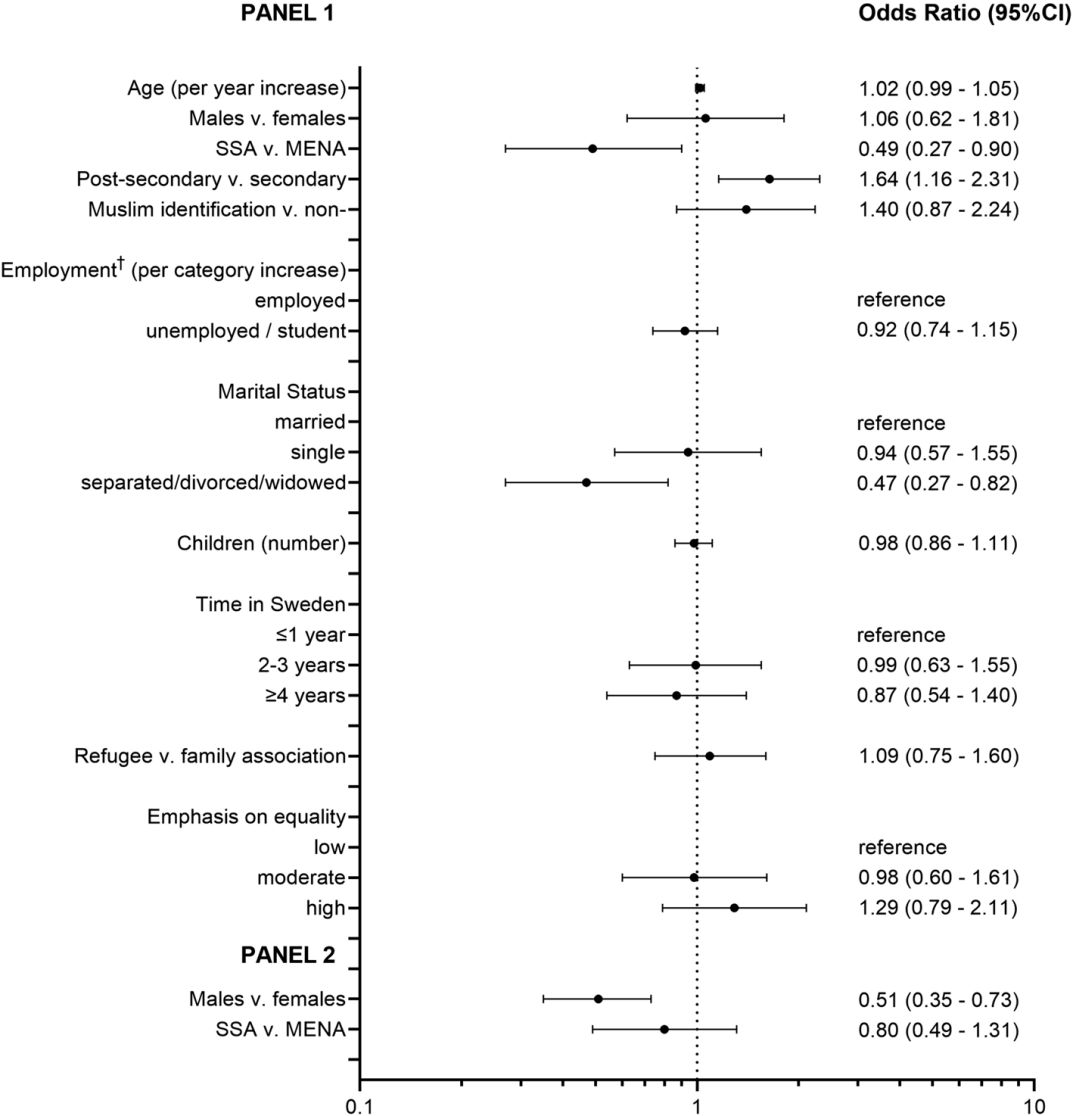
Partial proportional odds model using three levels^*^ of reproductive agency ^*^The lowest category of reproductive agency is the reference level. The first panel contrasts category 1 (low values of reproductive) with categories 2 (moderate values of reproductive agency) and 3 (high values of reproductive agency). The second panel contrasts categories 1 and 2 with 3. Odds ratios greater than one indicate that it is more likely that participants with the explanatory variable will be in a higher category. ^†^ORs for employment are per unit increase in category. The reference category is being employed, the second category ‘unemployed’, and the third category ‘student’

Of 4669 eligible respondents, 1372 (29.4%) had a value for reproductive agency. Almost three-quarters of participants (73.3%) reported a high degree of reproductive agency, 17.7% moderate, and 9.0% low. Similar to CSI values, most socio-demographic variables were associated with reproductive agency in the bivariate (unadjusted) analysis, apart from time lived in Sweden (Table 1). In the proportional odds model, the variables sex and region (MENA v. SSA) did not meet the proportional odds assumption, therefore, we fitted a partial proportional odds model for reproductive agency. There were no differences by sex when the lowest level of reproductive agency was compared to the two higher levels of reproductive agency. However, when low and moderate categories were combined and compared to the highest category of reproductive agency, men were less likely to report high reproductive agency compared to women (aOR 0.51, 95% CI: 0.35–0.73) (Fig. 2). Those with post-secondary education were more likely to report higher reproductive agency than respondents with less education (aOR 1.64, 95% CI: 1.16–2.31). High reproductive agency was negatively associated with being from SSA (OR 0.49, 95% CI: 0.26–0.90), and being separated, divorced, or widowed (aOR 0.47, 95% CI: 0.27–0.82) compared to those who were married (Fig. 2). Identifying as Muslim had no association with one’s sense of reproductive agency. Data on reproductive agency was missing for 70.6% of respondents. Missingness was not associated with age or sex; however, it was associated with level of education, region, and reason for moving to Sweden (additional file 4).

**Table 1:** Bivariate (unadjusted) associations between sociodemographic characteristics^a^ and reproductive agency

**Characteristic**	**Total (*N* = 1372)**	**Low reproductive agency** **n (%)**	**Moderate reproductive agency** **n (%)**	**High reproductive agency** **n (%)**	***P*-value**
**Total**		124 (9.0)	243 (17.7)	1005 (73.3)	
**Age, mean (SD), years**		29.70 (8.93)	30.95 (9.35)	32.26 (8.71)	< 0.001^*^
**Sex**					
Female	765 (55.9)	64 (51.6)	93 (38.6)	608 (60.6)	< 0.001^†^
Male	604 (44.1)	60 (48.4)	148 (61.4)	396 (39.4)	
**Region of birth**					
MENA	1181 (86.1)	91 (73.4)	216 (88.9)	874 (87.0)	< 0.001^†^
SSA	191 (13.9)	33 (26.6)	27 (11.1)	131 (13.0)	
**Religious identity**					
Muslim	1120 (81.6)	88 (71.0)	202 (83.1)	830 (82.6)	0.006^†^
Non-Muslim	252 (18.4)	36 (29.0)	41 (16.9)	175 (17.4)	
**Education**					
≤ Secondary	456 (39.1)	51 (50.5)	91 (46.7)	314 (36.1)	0.001^†^
Post-secondary	709 (60.9)	50 (49.5)	104 (53.3)	555 (63.9)	
**Employment**					
Employed	208 (18.2)	14 (13.2)	47 (24.7)	147 (17.5)	0.069^†^
Unemployed	198 (17.3)	18 (17.0)	25 (12.6)	155 (18.5)	
Student	737 (64.5)	74 (69.8)	126 (63.6)	537 (64.0)	
**Marital status**					
Married / co-habiting	904 (66.6)	70 (56.9)	137 (57.8)	697 (69.9)	< 0.001^†^
Single	354 (26.1)	37 (30.1)	82 (34.6)	235 (23.6)	
Separated / divorced / widowed	99 (7.3)	16 (13.0)	18 (7.6)	65 (6.5)	
**Children, mean (SD), number**		1.43 (1.47)	1.78 (1.95)	1.84 (1.79)	0.002^*^
**Time in Sweden, median (IQR) (years)**		3 (2–4)	3 (2–4)	3 (2–4)	0.518^‡^
≤ 1 year	330 (24.1)	29 (23.4)	54 (22.3)	247 (24.6)	0.364^†^
2–3 years	550 (40.1)	44 (35.5)	109 (45.0)	397 (39.5)	
≥ 4 years	491 (35.8)	51 (41.1)	79 (32.6)	361 (35.9)	
**Reason for moving to Sweden**					
Family association	504 (36.7)	41 (33.1)	74 (30.5)	389 (38.7)	0.038^†^
Refugee	868 (63.3)	83 (66.9)	169 (69.5)	616 (61.3)	
**Emphasis on equality**					
Low	182 (15.1)	21 (22.1)	32 (15.4)	129 (14.3)	0.016^†^
Moderate	446 (37.0)	44 (46.3)	79 (38.0)	323 (35.8)	
High	577 (47.9)	30 (31.6)	97 (46.6)	450 (49.9)	

^*^ANOVA was used to determine the *P *value

^†^Pearson χ^2^ test was used to determine the *P *value

^‡^Kruskall-Wallis test was used to determine the P value

^a^Data not available for all individuals. Missing values: gender 3 (3 participants identified as other); education, 207; employment, 229; marital status, 15; number of children, 14; time in Sweden, 1; equality, 167

### Association between socio-demographic characteristics and CSI values

**Fig. 3:**
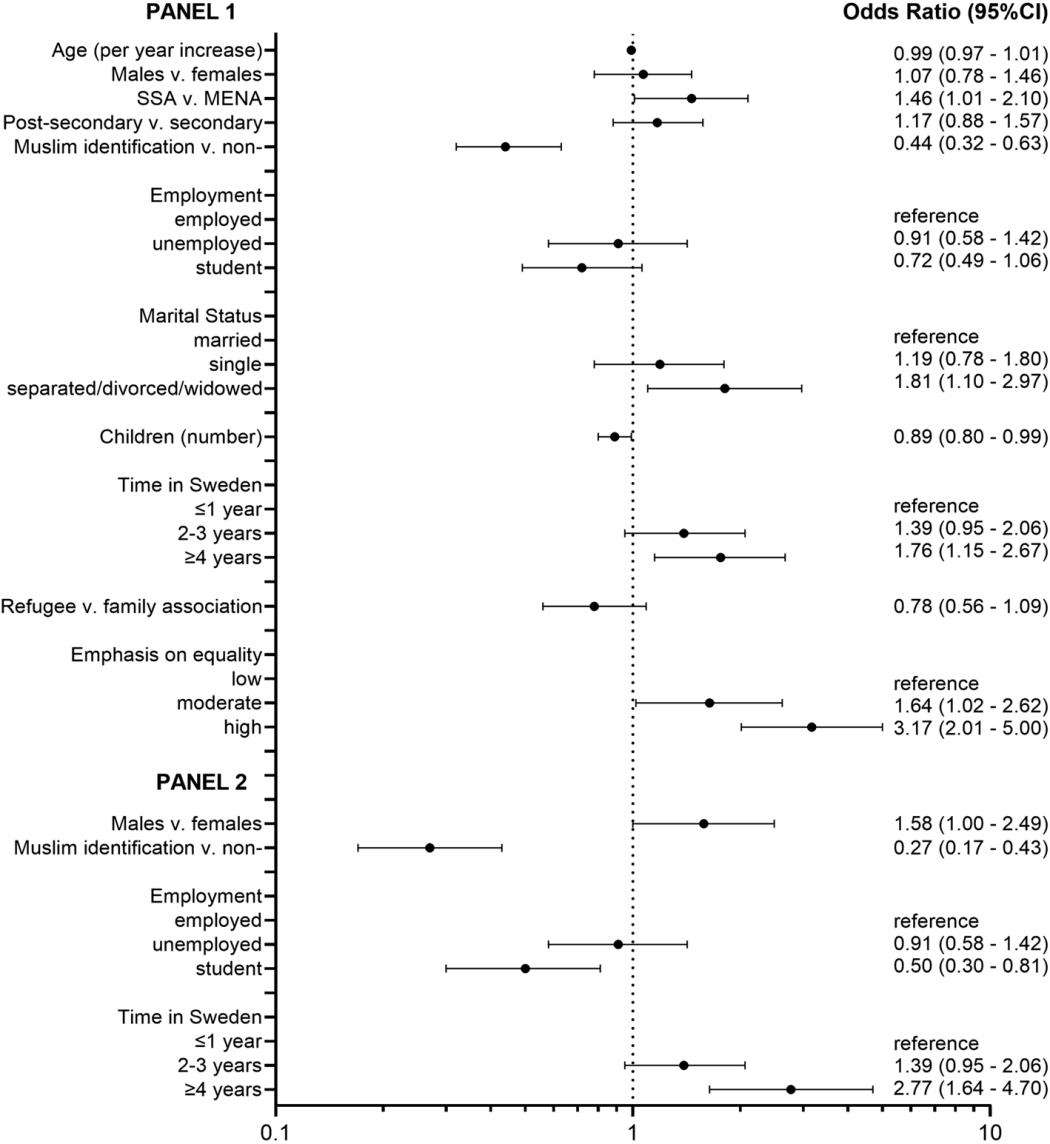
Partial proportional odds model using three levels^*^ of the Choice Sub-Index (CSI) ^*^The lowest category of the CSI is the reference level. The first panel contrasts category 1 (low values on the CSI) with categories 2 (moderate values on the CSI) and 3 (high values on the CSI). The second panel contrasts categories 1 and 2 with 3. Odds ratios greater than one indicate that it is more likely that participants with the explanatory variable will be in a higher category

Of 4669 eligible respondents, 2428 had a CSI value. Most participants had a low value on the CSI (78.8%), while only 4.0% had a high value on the CSI, i.e., most did not find divorce/abortion/homosexuality acceptable. The bivariate (unadjusted) analysis indicated that all factors, apart from having a post-secondary education and reason for migrating to Sweden, were associated with CSI values (Table 2). The likelihood ratio test led us to reject the proportional odds assumption. The Brant test revealed that some of the variables violated the parallel odds assumption of the proportional odds model, so we used a partial proportional odds model instead. This allowed the effects of the constrained variables to be fixed for level of the CSI, while sex, identification as Muslim, employment, and time lived in Sweden were allowed to differ. When the lowest category of the CSI was compared to the two higher categories, there was no association with sex (aOR 1.07, 95% CI: 0.78–1.46) (Fig. 3); however, when the two lower categories were compared to the highest category of the CSI, males may be more likely to have the highest value on the CSI compared to females (aOR 1.58, 95% CI: 1.00–2.49) (Fig. 3).

**Table 2:** Bivariate (unadjusted) associations between sociodemographic characteristics^a^ and the Choice Sub-Index (CSI)^b^

**Characteristic**	**Total (*N*** **= 2428)**	**Low value on the CSI** **n (%)**	**Moderate value on the CSI** **n (%)**	**High value on the CSI** **n (%)**	***P*-value**
**Total**		1914 (78.8)	326 (7.0)	188 (4.0%)	
**Age, mean (SD), years**		32.28 (8.9)	30.58 (9.0)	30.89 (10.0)	< 0.001^*^
**Sex**					
Female	1316 (54.3)	1054 (55.1)	178 (54.8)	84 (45.2)	0.033^†^
Male	1107 (45.7)	858 (44.9)	147 (45.2)	102 (54.5)	
**Region of birth**					
Middle East and North Africa	1969 (81.1)	1598 (83.5)	251 (77.0)	120 (63.8)	< 0.001^†^
Africa	459 (18.9)	316 (16.5)	75 (23.0)	68 (36.2)	
**Religious identification**					
Muslim	1805 (74.3)	1489 (77.8)	225 (69.0)	91 (48.4)	< 0.001^†^
Non-Muslim	623 (25.7)	425 (22.2)	101 (31.0)	97 (51.6)	
**Education**					
≤ Secondary	953 (46.3)	786 (46.9)	104 (40.9)	63 (49.2)	0.163^†^
Post-secondary	1104 (53.7)	889 (53.1)	150 (59.1)	65 (50.8)	
**Employment**					
Employed	342 (17.6)	221 (14.6)	61 (22.6)	60 (36.8)	< 0.001^†^
Unemployed	395 (20.3)	323 (21.3)	44 (16.3)	28 (17.2)	
Student	1211 (62.2)	971 (64.1)	165 (61.1)	75 (46.0)	
**Marital status**					
Married / co-habiting	1546 (66.4)	1269 (69.4)	174 (55.6)	103 (55.4)	< 0.001^†^
Single	619 (26.6)	443 (24.2)	105 (33.5)	71 (38.2)	
Separated / divorced / widowed	162 (7.0)	116 (6.3)	34 (10.9)	12 (6.5)	
**Children, mean (SD), number**		1.96 (1.89)	1.43 (1.67)	1.30 (1.73)	< 0.001^*^
**Time in Sweden, median (IQR) (years)**		3 (2–4)	3 (2–4)	4 (3–6)	< 0.001^‡^
≤ 1 year	537 (22.2)	449 (23.5)	61 (18.8)	27 (14.4)	< 0.001^†^
2–3 years	1028 (42.4)	838 (43.9)	139 (42.8)	51 (27.3)	
≥ 4 years	857 (35.4)	623 (32.6)	125 (38.5)	109 (58.3)	
**Reason for moving to Sweden**					
Family association	815 (33.6)	645 (33.7)	102 (31.3)	68 (36.2)	0.510^†^
Refugee	1613 (66.4)	1269 (66.3)	224 (68.7)	120 (63.8)	
**Emphasis on equality**					
Low	384 (18.1)	335 (19.9)	30 (10.7)	19 (11.4)	< 0.001^†^
Moderate	861 (40.5)	722 (43.0)	93 (33.2)	46 (27.7)	
High	882 (41.5)	624 (37.1)	157 (56.1)	101 (60.8)	

^*^ANOVA was used to determine the *P *value

^†^Pearson χ^2^ test was used to determine the *P *value

^‡^Kruskall-Wallis test was used to determine the *P *value

^a^Data not available for all individuals. Missing values: gender (5 participants identified as other); education, 371; employment, 480; marital status, 101; number of children, 118; time in Sweden, 6; equality, 301

^b^The CSI is a sub-index based on how acceptable participants find divorce, abortion, and homosexuality

Respondents from SSA were more likely to have higher values on the CSI than those from MENA (aOR 1.46, 95% CI: 1.01–2.10). Those identifying as Muslim had lower odds of having high values on the CSI than those not identifying as Muslim (aOR 0.44, 95% CI: 0.32–0.63). (Fig. 3). In addition, living in Sweden for four or more years was associated with greater values on the CSI (aOR 1.76, 95% CI: 1.15–2.67) compared to those who had more recently arrived. Compared to participants who were married or cohabiting, being separated, divorced, or widowed was associated with a greater likelihood of high values on the CSI (aOR 1.81, 95% CI: 1.10–2.97). Those who placed a greater value on gender equality compared with those who did not also had a greater odds of higher values on the CSI. Being a student rather than being employed was only associated with decreased odds of a high value on the CSI when low and moderate categories on the CSI were combined and compared to the highest level on the CSI (aOR 0.50, 95% CI: 0.30–0.81) (Fig. 3).

Data was missing for 48.0% of respondents (i.e. they had data on none or only one of the variables that comprise the CSI). Missingness was not associated with sex or reason for moving to Sweden; however, it was associated with age, level of education, and region (additional file 5).

## Discussion

We found that approximately three-quarters of first-generation migrants from SSA and MENA in Sweden self-reported a high degree of reproductive agency (i.e., how much can you decide yourself when it comes to having children). At the same time, almost 80% of participants had a low value on the CSI, i.e., one’s acceptance of divorce, abortion, and homosexuality. Few of the factors we examined were strongly associated with reproductive agency. Expectedly, a post-secondary education was associated with a greater sense of reproductive agency, while being separated, divorced, or widowed was associated with a lower level of agency. Factors associated with greater reproductive agency were not necessarily correlated with a higher CSI score, as we found with region. Migrants from SSA were more likely to have a higher CSI score than those from MENA, while migrants from MENA were more likely to have a greater sense of reproductive agency. The top three countries of origin of respondents from MENA are more predominantly Islamic than the top three countries of origin of respondents from SSA, however, after controlling for region, Muslim identification was the strongest factor associated with a lower value of the CSI. Other factors associated with higher levels on the CSI included living in Sweden for 4 years or longer, being either divorced, widowed or separated, and having a higher score on the equality index.

Sex was associated with reproductive agency insofar as males reported lower reproductive agency than females, but only when low and moderate categories were combined and contrasted with the highest category of reproductive agency. It is possible that males migrating from dominant patriarchal societies to a host country with greater gender equality could experience a diminished sense of reproductive agency. Such a hypothesis could be understood in the context of changing gender relations post-migration. For example, in a Canadian study, Ethiopian men undertook more household responsibilities, while women’s employment outside of the home largely increased. Resistance to these changes was most evident in men, as they felt their previously held power was undermined. However, over time, a shift occurred, resulting in the establishment of routines that diminished the gendered division of labour within the family [34].

We found that migrants with post-secondary schooling had greater odds of reporting a higher sense of reproductive agency compared to migrants with a lower level of education. Higher levels of education often lead to increased opportunities, greater economic stability, and social influence. When individuals have access to education, healthcare services, and economic stability, they are better equipped to make informed decisions about their sexual and reproductive health [35]. Therefore, increased social and economic opportunities benefit both men and women by providing them with the means to have control over their reproductive health and actively participate in this process [35].

The overall low values on the CSI may in part be explained by contextual factors in the countries of origin with regards to the components of the CSI. In MENA, divorce laws are predicated on highly gendered roles within a marriage and shari’a-based legal consequences, with conservatives opposing reforms to family law [36]. In countries where divorce is uncommon, it may be perceived as immoral and lead to social isolation and discrimination. Most abortion laws in MENA and SSA are punitive, with many countries only permitting abortion if the mother’s life is in danger [37, 38]. There is greater regional variation between MENA and SSA in the acceptance of divorce. In some parts of SSA where divorce is prevalent, it tends to be more socially acceptable [39]. Homosexuality remains criminalized in the countries from which the majority of the study participants originated, such as Syria, Afghanistan, Eritrea, and Somalia [40]. Individuals who identify as LGBTQIA + lack legal protections against discrimination and often face the threat of violence and persecution in these countries [40]. Consequently, migrants may hold more traditional viewpoints that inform their acceptance of homosexuality, thereby setting them apart from social and gender norms prominent in host countries like Sweden [41]. Although migrants represent a self-selected sample of their home country population, they may still carry the norms and values of their original societies [33].

Another factor that may contribute to the low values on the CSI is limited access to sexual education and lack of awareness regarding different reproductive rights in participants’ origin countries [42]. This is particularly pertinent to the sub-index’s abortion component. A lack of comprehensive sexual health education may restrict participants’ knowledge and understanding of sexuality and reproduction, potentially contributing to a narrower perspective and reinforcing traditional norms and beliefs, especially among migrant parents [43]. Such a limited understanding is reflective of a broader issue in that migrants often lack understandable information about the healthcare system, not only how it is organized and the services offered, but also why these services are offered in such a way, and their rights to healthcare in resettlement communities [44, 45].

We found no significant differences between male and female values on the CSI. Previous studies using WVS data have found that females are more accepting of abortion than males [46], and that males are more likely than females to perceive homosexuality as morally wrong [47]. Gender disparities related to household income and the risk of poverty suggest that women may face disadvantages in terms of financial losses during divorce, underscoring the likelihood that men are more accepting of divorce [48] These divergent views on the individual components of the CSI may explain why we found no overall difference between migrant men and women in our study population.

In Europe, there is a clear inverse relationship between age and people’s acceptance of abortion, homosexuality, and divorce [42]. We also expected that younger migrants would have a higher values on the CSI; however, we found no difference with age, and this may be explained in part because our study included only adults of reproductive age. While we included age and time in lived in Sweden as variables, we did not include age at which migrants moved to Sweden. If migrants moved to Sweden during their impressionable years i.e. adolescent and early adulthood, they may have been more susceptible to attitude change than those who moved when they were older [49]. There is also a lack of research specifically on how migrants’ sense of reproductive agency and values change over their lifespan. Instead, most studies focus on migrants’ access to sexual and reproductive health services, commodities, and information, in which it has been found that adolescent girls are at greater risk for lack of access compared to women [43]. Furthermore, studies tend not to disaggregate by age, making comparisons challenging.

Globally, Sweden is positioned at the high end of emancipated values. As individuals migrate from societies characterized by authoritarian values, often intertwined with closed and patriarchal religious structures, they may carry these values with them [27]. Migrating to Sweden and experiencing a cultural shift, which is often defined as acculturation (the process of adapting to the culture of a new host country) [50], can have a profound impact on the values and beliefs of migrants as they become exposed to new normative contexts relating to SRHR with a more open and egalitarian cultural environment. We found that the longer migrants lived in Sweden, the greater their acceptance of a combined measure of divorce, abortion and homosexuality. Our findings are consistent with evidence that migrants’ values change over time in their new destination, during which migrants are exposed to and become familiarized with the destination country’s social norms [26]. This has also been found in previous studies, for example, results from the 2019 Swedish MWVS report showed that migrants from seven selected countries living in Sweden since 2010 had greater values on the CSI compared to migrants who had been living in Sweden since 2018 [27].

Ultimately, reproductive agency primarily centers on individuals’ perceptions in making decisions about certain aspects of their reproductive health, whereas the CSI assesses the acceptance of homosexuality, abortion, and divorce. Both perspectives capture different facets of this complex issue and are vital to our understanding of reproductive decision-making and migrants' SRHR values. In addition, other implicit norms influence SRHR, such as social hierarchy and political beliefs, and these are frequently overlooked or unacknowledged when migrants resettle in a new country [51]. Placing greater emphasis on integration in the resettlement process can have several benefits for migrants and healthcare providers. For example, it allows for a better understanding of diverse cultures and healthcare practices, which promotes more inclusive and culturally sensitive care, fostering mutual understanding [52]. If integration programmes for newly arrived migrants in Sweden and other European countries [53] work towards closing communication gaps in healthcare, improving citizenship and belonging, and also promoting knowledge among migrants with respect to SRHR laws and policies, it may contribute to higher CSI values and a greater sense of reproductive agency [54].

To the best of our knowledge, this is the first study to examine both perceived reproductive agency and the acceptance of divorce, abortion and homosexuality among adult first-generation migrant men and women in Europe. The strengths of this study include using a validated index of values, which has proven reliable in different cultural and national settings [55]. A limitation of our study is we grouped migrants into two regions, neither of which is homogeneous. Countries vary greatly in social and gender norms, and social and economic policies. Other limitations include that the study relies on self-reported data, which may have provoked socially desirable responses. We also used a single-item question versus a more comprehensive index to assess reproductive agency. We used purposive rather than random sampling to select municipalities in Sweden, therefore, we are unable to draw statistical inferences from the sample. While the aim of our sampling procedures was to produce a representative population, there may be some discrepancies in characteristics between the migrant population in Sweden as a whole and our study population [32], which may have affected our results.

Despite a large sample size, a limitation of our study is missing data, a factor to be considered when assessing the generalisability of our findings and which may have also biased our results. Missing data could be attributed to participant fatigue, as the survey was lengthy. It is also possible that participants felt reluctant to answer certain questions related to abortion, homosexuality, and reproductive agency due to the sensitive nature of these topics [56]. The degree of missingness was not associated with sex; however, participants from MENA were more likely to have outcome data than those from SSA, as were those with a higher level of education. Higher levels of education are associated with greater reproductive agency [57] and acceptance of the components of the CSI [17], potentially inflating our results. At the same time, having more outcome data from MENA respondents may have skewed our results on CSI values in the opposite direction, as migrants from MENA have been found to be less accepting than those from SSA of some of the components of the CSI.

## Conclusions

Our study found that first-generation migrant adults of reproductive age from MENA and SSA to Sweden expressed a high level of reproductive agency but had low scores on the CSI. This indicates that while migrants express an ability to make decisions regarding how many, when, and with whom to have children, they find the justifiability of divorce, abortion, and homosexuality less acceptable. The longer migrants had lived in Sweden was associated with greater acceptance, suggesting that individuals may express more emancipative values as they spend more time in Sweden, Our findings could be integrated into introduction programmes and policies for newly arrived migrants. Integration programs may benefit from including information and opportunities to reflect on norms related to SRHR and gender equality and clarify their link to human rights and Swedish laws. These efforts can help bridge gaps in reproductive rights awareness and promote equitable access to sexual and reproductive health services for all residents.

## Supplementary Information

Supplementary Material 1.

## Data Availability

The dataset is available is available from Bi Puranen and the corresponding author, Elin C. Larsson.
