# Supplementary material for: Reproductive agency and the acceptability of divorce, abortion, and homosexuality among migrants from the Middle East and Africa living in Sweden–a cross-sectional analysis
Source: Int J Equity Health. 2025 Feb 24;24:53. doi: 10.1186/s12939-025-02400-x (PMC11853754; doi:10.1186/s12939-025-02400-x)
Supplement: Supplementary file 1 — Supplementary Material 1. [file 12939_2025_2400_MOESM1_ESM.docx]

**Supplementary Online Material**

**Additional file 1: Description of sampling, sample and data collection and**

1. **Introduction**
2. **Sampling procedure**
3. **Missing data**
4. **Consequences for the representativity**
5. **Introduction**

Interviewing migrants creates certain obstacles that are not present when interviewing people in their countries of origin. These obstacles mainly revolve around the situations in which the interviews are conducted, rather than being about sampling procedures. Two key factors are the level of motivation on the part of the participant and the level of perceived insecurity when sensitive questions are asked.

The sampling procedure within the World Values Survey is described below and normally conducted through face-to-face interviews using the language preferred by the respondent. Migrant World Values Survey (MWVS) must use another sampling procedure, as the respondents do not live in their countries of origin and in a different cultural context than where they were born.

1. **Sampling procedure**

## PHASE I:

Step 1: Initially, MWVS used the full population register from the Bureau of Statistics for Sweden, including all people registered as living in Sweden.

Step 2: Everyone born in Sweden was omitted.

Step 3: Everyone born in a Western country (22 countries that are members of the European Union; Norway, Switzerland, the North American continent; Australia and New Zealand were omitted.

Step 4: A full sample including all registered migrants born in a non-Western country was created. The sample was thus drawn from the total number of non-Western migrants registered in Sweden who lived in certain municipalities.

## PHASE II:

**Sampling of municipalities:**

MWVS used the system for selecting municipalities developed by Municipalities Assemblies in European Local Governance (MAELG) for Sweden. This is an EU based system aiming to determine principles for stratifying municipalities by population size and structure, region, and economic structure. The sample criteria were:

- Percentage of residents born in non-European countries,
- The level of unemployment,
- Election results,
- Type of municipality (urban/rural and size).

In total, 54 municipalities out of the 290 existing in Sweden were drawn using the MAELG list, representing 20 percent of all non-Western migrants in Sweden.

## PHASE III:

**Sampling of migrants within the 54 municipalities:**

Three selection modes were chosen:

Participants were gathered via three sampling methods in the municipalities:

- 1. Respondents interviewed via SFI (Swedish for foreigners) which was 4 327 respondents or 66% of the full sample,
  2. Respondents interviewed within the ordinary school system (1 207 or 19% of the full sample.
  3. Respondents invited to take part via a personal invitation (982 or 15%).

As for the interviews made via SFI, we randomly picked one class in each level (there are four levels in the system). Within each of these classes, we interviewed everyone. They had the possibility of declining but very few did so; see the absence report (Puranen 2019, appendix table B2). Out of 7 302 respondents who fulfilled the participation criteria, 7 188 agreed to participate (114 declined). The number of participants with missing data on country- of-origin was 466 and the number of participants with missing data on the number of years spent in Sweden was 786. As these participants were excluded from further analysis, the total primary loss was 786, or 11 % of the original sample (see figure 1). The total sample included in the study was 6 516.

To also include people who have stayed longer periods in Sweden (who did not take part in SFI or the ordinary school system), we invited respondents to take part via a postal invitation. They came to the local library where we interviewed them or if they chose some other place. Thus, we obtained a representative sample also including time spent in Sweden (see table 1, manuscript).

The MWVS questionnaires were administered face-to-face across Sweden between September 19, 2018, and November 27, 2019. Participants completed the questionnaire in the municipality in which they were registered and in the language with which they were most comfortable (Arabic, Dari, English, Somali, Swedish, Tigrinya, or Turkish).

1. **Missing data**

The number of respondents answering the choice index variables was *n* = 2 428 and the number of respondents with a response to reproductive agency was *n* = 1 372. The recommended lowest level of number of interviews within the WVS are 1200 per country. Data on the variable reproductive agency was missing for 70.6% of respondents. Missingness was not associated with age or sex; however, it was associated with level of education, region, and reason for moving to Sweden (Additional file 3). The number of respondents missing a response was greater for refuges than for migrants arriving via family association. The number of missing values was also greater for respondents with a lower level of education. The missing data respondents came mainly from Sub-Saharan Africa while the number of missing data on reproductive agency from the MENA region was substantially lower. There are many explanations for respondents missing information on some variables:

- - The main reason for missing data was the time factor. The time given for each interview was limited to 90 minutes. Illiterate respondents sometimes could not complete the questionnaire within that time. The time it takes to read and answer the questions also differs between languages. Tigrinya, the language spoken in Eritrea, is more time-consuming than for example English.
  - A second reason might be that the questionnaire was only translated to the languages spoken by more than 15 percent of the population in the most common non-Western migrant countries (Arabic, English, Tigrinya, Somali, Dari, Pashtun, Turkish and Swedish) Migrants speaking other languages might therefore find it more challenging to complete the full questionnaire and the staff present could not help the respondents either due to lack of knowledge on the specific language.
  - A third reason for missing out on some variables could be that the respondent felt uncomfortable answering some of the questions.
  - When studying migrant populations with low levels of education this also should be considered when assessing the generalizability of the findings. Approximately 75 percent of the respondents missing values on reproductive agency have a low level of education.

These factors may explain the level of missing data on some variables in the sample.

1. **Consequences for the representativity**

The MWVS tested the generalizability of the findings by interviewing the non-Western migrants in all municipalities within a region (*n* = 10 986). This test was made in three regions and the results were analyzed by calculating cleavages. We can conclude that the results from the regional sample did not deviate from the initial national sample. In the national sample used in this article, the value was 0,454. For the regional sample with all municipalities within a region, the value was 0,456. From this we draw the conclusion that the national sample of 54 municipalities who represented 20 percent of all non-Western migrants is representative. These descriptive analysis is unfortunately only presented in Swedish, but there are four reports describing the results from both the initial sample used here and the additional regional samples with an English summary in each of them:

Results drawn from the sample are presented in a Swedish report series:

Puranen, B. (2019). *With the voice of the migrants, Part 1: The subjective integration*. Institute for Future Studies, Stockholm.

Puranen, B. (2021). *With the voice of the migrants, Part 2: How to become a Värmlänning - Identity transfer*. Institute for Future Studies, Stockholm.

Puranen, B. (2023). *With the voice of the migrants, Part 3: How do values change over time*. Institute for Future Studies, Stockholm.

Puranen, B. (2024). *With the voice of the migrants, Part 4: Equality and the concept of work*. Institute for Future Studies, Stockholm.

**Additional file 2: Participants by country of origin**

| **SSA** | **Number of participants** | **MENA** | **Number of participants** |
| --- | --- | --- | --- |
|  |  |  |  |
| Angola | 1 | Afghanistan | 507 |
| Burundi | 10 | Algeria | 9 |
| Cameroon | 4 | Palestine | 58 |
| D.R. Congo | 32 | Iran | 127 |
| Ethiopia | 86 | Iraq | 369 |
| Eritrea | 501 | Israel | 1 |
| Gambia | 6 | Jordan | 21 |
| Ghana | 13 | Kuwait | 24 |
| Guinea | 1 | Lebanon | 54 |
| Côte d’Ivoire | 6 | Libya | 21 |
| Kenya | 19 | Morocco | 41 |
| Namibia | 1 | Qatar | 1 |
| Nigeria | 31 | Saudi Arabia | 34 |
| Madagascar | 1 | Syria | 2047 |
| Rwanda | 2 | United Arab Emirates | 16 |
| Senegal | 2 | Tunisia | 15 |
| Sierra Leone | 5 | Turkey | 130 |
| Somalia | 344 | Egypt | 27 |
| South Africa | 8 | Yemen | 19 |
| Sudan | 57 |  |  |
| Uganda | 10 |  |  |
| Burkina Faso | 1 |  |  |
| Zambia | 3 |  |  |
| Zimbabwe | 1 |  |  |
| Tanzania | 3 |  |  |

**Additional file 3. Baseline characteristics**

| **Characteristic^*^** | **Total (N=4669)** |
| --- | --- |
| **Age, mean (SD), years** | 31.47 (9.10) |
| **Gender** |  |
| Female | 2515(53.9) |
| Male | 2142 (45.9) |
| Other | 12 (0.3) |
| **Region of birth** |  |
| Middle East and North Africa | 3521 (75.4) |
| Sub-Saharan Africa | 1148 (25.6) |
| **Religious identity** |  |
| Muslim | 2940 (63.0) |
| Non-Muslim | 1729 (37.0) |
| **Education** |  |
| ≤Secondary | 1810 (49.7) |
| Post-secondary | 1830 (50.3) |
| **Employment** |  |
| Employed | 546 (16.9) |
| Unemployed | 617 (19.1) |
| Student | 2069 (64.0) |
| **Marital status** |  |
| Married / co-habiting | 2546 (63.0) |
| Single | 1206 (29.8) |
| Separated | 54 (1.3) |
| Divorced | 182 (4.5) |
| Widowed | 53 (1.3) |
| **Children, mean (SD), number** | 1.75 (1.87) |
| **Time in Sweden, median (IQR) (years)** | 3 (2-4) |
| ≤1 year | 1039 (22.3) |
| 2-3 years | 2029 (43.6) |
| ≥4 years | 1583 (34.0) |
| **Reason for moving to Sweden** |  |
| Family association | 1533 (32.8) |
| Refugee | 3136 (67.2) |
| **Emphasis on equality** |  |
| Low | 510 (17.3) |
| Moderate | 1148 (39.0) |
| High | 1289 (43.7) |

**^*^**Data not available for all individuals. education, 1029 (22.0%); employment, 1437 (30.8%); marital status, 628 (13.5%); number of children, 666 (14.2%); time in Sweden, 18 (0.4%); equality, 1722 (36.9%).

**Additional file 4: Characteristics of participants with a response to the variable reproductive agency v. those missing a response to the variable reproductive agency**

| **Characteristic** | **Respondents with a response to reproductive agency (n=1372)** | **Respondents missing a response to reproductive agency (n=3297)** | **Total**  **(n=4669)** | **p-value** |
| --- | --- | --- | --- | --- |
| **Age (years)** | n, % | n, % |  | 0.120 |
| 18-24 | 349 (27.1) | 941 (72.9) | 1290 |  |
| 25-30 | 288 (30.6) | 654 (69.4) | 942 |  |
| 31-40 | 463 (30.9) | 1034 (69.1) | 1497 |  |
| >40 | 272 (28.9) | 668 (71.1) | 940 |  |
| **Sex** |  |  |  | 0.098 |
| Female | 765 (30.4) | 1750 (69.6) | 2515 |  |
| Male | 604 (28.2) | 1538 (71.8) | 2142 |  |
| **Education** |  |  |  | <0.001 |
| ≤Secondary | 456 (25.2) | 1354 (74.8) | 1810 |  |
| Post-secondary | 709 (38.7) | 1121 (61.3) | 1830 |  |
| **Region** |  |  |  | <0.001 |
| MENA | 1181 (33.5) | 2340 (66.5) | 3521 |  |
| SSA | 191 (16.6) | 957 (83.4) | 1148 |  |
| **Reason for moving to Sweden** |  |  |  | <0.001 |
| Family association | 504 (32.9) | 1029 (67.1) | 1533 |  |
| Refugee | 868 (27.7) | 2268 (72.3) | 3136 |  |

**^*^**Data not available for all individuals. Missing values: gender (12 participants identified as other); education, 1029.

**Additional file 5: Characteristics of participants with the *choice* index v. those missing the *choice* index**

| **Characteristic** | **Respondents with the *choice index***  **(n=2428)** | **Respondents missing the *choice* index**  **(n=2241)** | **Total**  **(n=4669)** | **p-value** |
| --- | --- | --- | --- | --- |
| **Age (years)** | n, % | n, % |  | 0.007 |
| 18-24 | 620 (48.1) | 670 (51.9) | 1290 |  |
| 25-30 | 496 (52.7) | 446 (47.3) | 942 |  |
| 31-40 | 797 (53.2) | 700 (46.8) | 1497 |  |
| >40 | 515 (54.8) | 425 (45.2) | 940 |  |
| **Sex** |  |  |  | 0.660 |
| Female | 1316 (52.3) | 1199 (47.7) | 2515 |  |
| Male | 1107 (51.7) | 1538 (48.3) | 2142 |  |
| **Education** |  |  |  | <0.001 |
| ≤Secondary | 953 (52.7) | 857 (47.3) | 1810 |  |
| Post-secondary | 1104 (60.3) | 726 (39.7) | 1830 |  |
| **Region** |  |  |  | <0.001 |
| MENA | 1969 (55.9) | 1552 (44.1) | 3521 |  |
| SSA | 459 (40.0) | 689 (60.0) | 1148 |  |
| **Reason for moving to Sweden** |  |  |  | 0.267 |
| Family association | 815 (53.2) | 718 (46.8) | 1533 |  |
| Refugee | 1613 (51.4) | 1523 (48.6) | 3136 |  |

**^*^**Data not available for all individuals. Missing values: gender (12 participants identified as other); education, 1029.
